# Complementary and Alternate Management of Glaucoma: The Verdict so Far

**DOI:** 10.5005/jp-journals-10008-1161

**Published:** 2014-06-12

**Authors:** Shibal Bhartiya, Parul Ichhpujani

**Affiliations:** Consultant, Department of Ophthalmology, Glaucoma Services, Fortis Memorial Research Institute, Gurgaon, Haryana, India; Assistant Professor, Department of Ophthalmology, Glaucoma Services, Government Medical College and Hospital, Chandigarh, India

**Keywords:** Acupuncture, Alternative therapy, Complementary therapy, Ginkgo biloba, Glaucoma, Yoga.

## Abstract

Complementary and alternative medicine deserves scientific scrutiny as patients with glaucoma often lose vision despite adequate medical or surgical treatment. Most glaucomatologists abstain from recommending alternative medicine as there is little evidence to support most of the recommendations for complementary and alternate management (CAM) use in glaucoma. Megavitamin supplementation has not been shown to have a long-term beneficial effect on glaucoma. In a glaucomatous eye, a very modest benefit of IOP-lowering may be offset by the temporary elevation in IOP that accompanies exercise. There is little evidence to support the use of special diets, acupuncture, relaxation techniques, or therapeutic touch for the treatment of glaucoma. Marijuana can have a profound lowering of IOP, but the low response rate, short half-life, and significant toxicity are strong indicators that it is not an appropriate therapeutic agent. Future research must be carried out to document the effect of CAM not only on IOP, but also on perimetric tests or other objective parameters, such as ocular blood fow and nerve fiber layer thickness.

**How to cite this article:** Bhartiya S, Ichhpujani P. Complementary and Alternate Management of Glaucoma: The Verdict so Far. J Curr Glaucoma Pract 2014;8(2):54-57.

## INTRODUCTION

The National Center for Complementary and Alternative Medicine defines alternative medicine as ‘… those treatments and healthcare practices not taught widely in medical schools, not generally used in hospitals, and not usually reimbursed by medical insurance companies.'^1^ For practical considerations, all the disease management strategies other than conventional therapy, i.e. pharmaceutical, laser, or surgical treatment known to lower intraocular pressure (IOP) are included in the purview of complementary and alternate management (CAM) of glaucoma. Increasing insights into glaucoma pathophysiology have opened newer vistas of therapeutic approaches, most of which are as yet empirical or investigational, including agents that can improve blood fow to the eye and reduce oxidative stress. With increasing emphasis on lifestyle modifications for combating disease, and a holistic approach to a healthier body, more and more people, patients and doctors alike, have been found to be receptive of CAM. In fact, as many as 5 to 10% of glaucoma patients reportedly use CAM, with almost 60% using more than one modality.^2^ Interestingly one study reported that more than 60% of patients using CAM do not divulge these details to their doctor, while another reported that 72% of these patients did actually discuss details with their treating ophthalmologist.^3^ This may potentially put the patients at risk, since the information sourced online and through peers is not always reliable, and the hazards of self-medication well-known. It is important, therefore that glaucomatologists offer advice to patients about the evidence and potential risks of CAM therapies.

This article aims to bring together the currently available evidence, most of which remains empirical and anecdotal in nature.

### Rationale

The IOP-glaucoma-optic nerve damage paradigm remains open ended for now. Almost 90% of patients with elevated IOP never develop damage to the optic nerve, while more than one-third of patients with POAG, never have documented elevations in IOP. Also, glaucomatous nerve damage has been known to progress despite good IOP control. There is sufficient evidence, therefore, to indicate that glauco-matous optic nerve damage is multifactorial, vascular-ischemic factors being an important contributory element.

Current glaucoma therapy, however, is only directed toward IOP reduction. Nonpressure-dependent risk factors are also getting considerable attention in glaucoma management, but the disease modifying capability of these approaches are yet to find widespread clinical application. CAM, for now, is an attempt to bridge this lacuna.

Complementary and alternate management becomes increasingly relevant for end stage glau comas, normal ten sion glaucomas, and those patients who continue to progress despite good IOP control. Glaucomas associated with vasospastic phenomenon are also potential candidates for CAM.

### Available Modalities

Rhee et al considered nine areas as complementary and alter native for glaucoma: herbal remedies, acupuncture, homeo pathy, faith healing, meditation, megavitamin therapy, therapeutic touch, exercise, and dietary modification (includes commercial weight loss programs and specialized diets, such as high in vitamin A, low cholesterol).^2^ However, for the purpose of this review, we would broadly classify the available CAM modalities into lifestyle modification, and supplementary therapeutic options.

Lifestyle Modification

*Exercise*: Aerobic exercise has been reported to cause lowering of IOP, while other authors have reported that isometric exercise like lifting weights may produce a small IOP increase during exertion.^4^ Lower levels of physical activity were associated with lower OPP.^5^ IOP spikes have been noted following rigorous exercise in pigmentary glaucoma, following a burst of pigment release.^6^

*Yoga and meditation*: Meditation is known to help patients cope with disease better and have a positive impact on their quality of life. Given that both the diagnosis and the management of glaucoma have a deleterious effect on the quality of life of the patient, it may be presumed that both yoga and meditation can help the patient cope with the disease better. However, it is essential to remember that there is evidence that certain yoga postures, especially the inverted position (like Shirshasana) can lead to an IOP spike.^7^

*Scuba diving and bungee jumping*: These adventure sports should be avoided by patients of glaucoma, especially advanced disease, because of resultant IOP spikes. Increased IOP has been explained with raised episcleral venous pressure or increased choroidal volume due to vascular engorge ment.^8^

*Stress*: The deleterious effects of stress are manifold, and glaucoma is no exception. Prolonged stress can result in endogenous cortisol and catecholamine release which may increase IOP.^9^ If a patient with stable disease develops a dramatic rise in IOP or deterioration of visual function, it may be prudent to ask them about potential psychosocial or environmental stress factors. Meditation, biofeedback and relaxation techniques may help manage stress better.

*Necktie*: A tight necktie may increase IOP in susceptible individuals by increasing the venous pressure. There is evidence that the IOP is restored to baseline by homeostatic mechanisms over 15 minutes.^10^

*Caffeine*: A typical cup of coffee may result in a 1 to 4 mm Hg rise in IOP that lasts for at least 90 minutes. It is therefore recommended that glaucoma patients restrict their consumption to less than 200 mg of caffeine per day.^11^

*Alcohol*: Alcohol consumption has been variably related to dose-related IOP reduction which may last for several hours through temporary osmotic effect. Beer or water consumption, in large quantities, is known to increase IOP significantly due to volume overload.^11^ Since red wine is rich in antioxidants, its role in neuroprotection has generated considerable interest. Its use may be advocated with the caveat that alcohol does increase the risk of liver disease.

*Musical instruments*: A person playing high wind musical instruments (e.g. Saxophone) can have two-fold increase in his IOP from baseline level for short duration.^12^

*Sleeping position*: How patients sleep at night may pose another preventable risk factor in glaucoma. An increase in IOP has been noted when patients are positioned in the supine position as compared to an upright position. These differences may be even more pronounced in patients already diagnosed with normal-tension glaucoma. A recent study found that an action as simple as having the patient sleep at a 30° incline may reduce night time IOP by up to 20% in some patients.^13^ Sleeping with two or three pillows may boost the effects of other glaucoma therapies.

Dietary Supplements

*Dietary anti-oxidants*: Red wine, dark chocolate, cocoa, green tea, curcumin, glutathione, n-6 to n-3 polyunsaturated fatty acid, are credited with anti-oxidant, and therefore potentially neuroprotective, abilities.^11^ There is however, scant evidence to prove their beneficial effect. Also, the quantities required to have a beneficial effect may be difficult to consume.

*Marijuana*: The reduction in IOP due to reduced aqueous production is attributed to Delta-9-tetrahydrocannabinoid found in marijuana. This IOP lowering is for short lived, and one needs to smoke marijuana every 3 hours for 24-hour IOP control.^14^ Additionally, marijuana may lower systemic blood pressure and could potentially decrease ocular perfusion. Since it has hallucinogenic and addictive predilection, its use in glaucoma therapy remains unacceptable till such time that an analog without these deleterious effects becomes commercially available.

*Gingko biloba*: The main components of the Gingko biloba leaf extract (GBLE) are favonoid glycosides and terpene lactones, and are credited with improving pre-existing visual field loss in some patients with normal tension glaucoma, by vasoregulatory mechanisms.^15^ Its multiple beneficial actions, reportedly include increased ocular blood fow, antioxidant activity, platelet activating factor inhibitory activity, nitric oxide inhibition, and neuroprotective activity. GBLE also has a mild blood-thinning effect, synergistic with aspririn, and may be a rheological modifer of blood fow. However, its use must be under medical supervision, especially on aspirin or warfarin analogs, so as to prevent bleeding diathesis.

*Lycium barbarum (Wolfiberry)*: Studies have shown that L barbarum extract may be a potential candidate for the development of neuroprotective drug against the loss of RGCs in glaucoma.^15^ Although the neuroprotective effect of L barbarum polysaccharides on the degeneration of retinal ganglion cells in ocular hypertension or the secondary dege neration of retinal ganglion cells after partial optic nerve transection has been shown histologically, its effect on preserving visual function is still uncertain.

*Bilberry*: Bilberry extracts contain high quantities of antho-cyanin, a favonoid with antioxidant properties and have received considerable attention in the recent past as an attractive adjuvant to conventional therapy.^11^

*Salt*: There is anecdotal evidence that supplementary salt can help combat vascular dysregulation, by an increase in syste mic blood pressure. This approach is contraindicated for patients with comorbidities like coronary artery disease which would be exacerbated by an increase in systemic blood pressure. Increasing blood pressure may be protective against the blood pressure ‘dips' that occur overnight, especially in normal tension glaucomas.^16^

*Omega-3 fatty acids*: Omega-3 fatty acids are credited with promotion of vascular health. There is anecdotal evidence that omega-3 FAs may have a therapeutic benefit in glaucoma patients, despite the mild reduction in systemic blood pressure. This may be attributed to a stabilization of ocular blood fow, and not increase or decrease in the blood pressure per se.^17^

*Vitamin C*: The anti-oxidant property of ascorbic acid has led to considerable interest in its possible role in glaucoma therapy. While low- and high-dose supplementary consumption of vitamin C has been variably found to be associated with decreased odds of glaucoma, serum levels of vitamin C do not correlate with glaucoma prevalence. Aside from a temporary osmotic effect from high dose intravenous ascorbic acid, there is no evidence that megavitamin supplementation has a beneficial effect on glaucoma.^3,18^

Alternate Systems of Medicine

It is impossible to draw reliable conclusions from available data to support the use of acupuncture or acupressure for the treatment of glaucoma.

*Acupuncture*: The underlying principle of acupuncture is that disorders related to the fow of Chi (the traditional Chinese

concept translated as vital force or energy) can be prevented or treated by stimulating the relevant points on the body surface. Its use in glaucoma however remains empirical, and with no evidence regarding its efficacy.^19^

*Ear acupressure*: Ear acupressure as a method of sen sory stimu lation has been proposed to have a role in neuro-protec tion through regulating nerve growth factor and brain-derived neurotrophic factor and their receptors, thereby encouraging the survival pathway in contrast to the pathway to apoptosis.^20^ Evidence to its efficacy, however, remains unavailable.

*Therapeutic touch*: Therapeutic touch is based on the ability of its practitioners to manually manipulate a patient's ‘human energy field.' A well-designed study of 12 therapeutic touch practitioners found that these practitioners were unable to detect an individual's energy field.^21^ There has been no study of the possible effect of therapeutic touch in patients with glaucoma.

## CONCLUSION

Because of ethical considerations, randomized control trials comparing CAM alone with standard glaucoma treatment or placebo are unlikely to be justified since conventional IOP lowering modalities are established standards of care. Clinical practice decisions regarding CAM therefore will have to rely on the judgment of the glaucomatologist as well as patient preferences, with communication between the two being key. It is incumbent upon the treating physician to emphasise to the patient that CAM is merely an adjuvant, and not a substitute for glaucoma medication, in the current scenario. The safety of CAM therapies is an important issue. There is widespread belief in the general public that CAM therapies are ‘all natural' and therefore harmless. However, commonly used alternative therapies can have significant adverse effects.
